# Spatio-temporal dynamics of cortical drive to human subthalamic nucleus neurons in Parkinson's disease

**DOI:** 10.1016/j.nbd.2018.01.001

**Published:** 2018-04

**Authors:** Andrew Sharott, Alessandro Gulberti, Wolfgang Hamel, Johannes A. Köppen, Alexander Münchau, Carsten Buhmann, Monika Pötter-Nerger, Manfred Westphal, Christian Gerloff, Christian K.E. Moll, Andreas K. Engel

**Affiliations:** a MRC Brain Network Dynamics Unit, Dept. of Pharmacology, University of Oxford, Oxford OX1 3TH, United Kingdom; b Department of Neurophysiology and Pathophysiology, University Medical Center Hamburg-Eppendorf, 20246 Hamburg, Germany; c Department of Neurosurgery, University Medical Center Hamburg-Eppendorf, 20246 Hamburg, Germany; d Department of Paediatric and Adult Movement Disorders and Neuropsychiatry, Institute of Neurogenetics, University of Lübeck, Lübeck, Germany; e Department of Neurology, University Medical Center Hamburg-Eppendorf, 20246 Hamburg, Germany

**Keywords:** Parkinson's disease, Subthalamic nucleus, Beta oscillations, Motor cortex, Neuronal synchronisation

## Abstract

Pathological synchronisation of beta frequency (12–35 Hz) oscillations between the subthalamic nucleus (STN) and cerebral cortex is thought to contribute to motor impairment in Parkinson's disease (PD). For this cortico-subthalamic oscillatory drive to be mechanistically important, it must influence the firing of STN neurons and, consequently, their downstream targets. Here, we examined the dynamics of synchronisation between STN LFPs and units with multiple cortical areas, measured using frontal ECoG, midline EEG and lateral EEG, during rest and movement. STN neurons lagged cortical signals recorded over midline (over premotor cortices) and frontal (over prefrontal cortices) with stable time delays, consistent with strong corticosubthalamic drive, and many neurons maintained these dynamics during movement. In contrast, most STN neurons desynchronised from lateral EEG signals (over primary motor cortices) during movement and those that did not had altered phase relations to the cortical signals. The strength of synchronisation between STN units and midline EEG in the high beta range (25–35 Hz) correlated positively with the severity of akinetic-rigid motor symptoms across patients. Together, these results suggest that sustained synchronisation of STN neurons to premotor-cortical beta oscillations play an important role in disrupting the normal coding of movement in PD.

## Highlights

• Multi-channel EEG with coincident STN single unit and local field potential recordings
• Variable time delays between beta oscillations in different cortical areas and STN neurons.
• Frontal/premotor cortical areas have most stable oscillatory synchronisation with STN neurons.
• Correlation between cortico-subthalamic beta-frequency synchronisation and clinical scores in PD.

## 1 Introduction

Synchronised oscillations in the cortico-basal ganglia network are a prominent feature of patients with Parkinson's disease (PD) and its animal models (Brown et al., 2001, Sharott et al., 2005, Tachibana et al., 2011). Oscillatory activity in the beta-range (broadly defined here as any frequency between 12 and 35 Hz) in the STN of PD patients is coupled to that in the cortex and basal ganglia output nuclei (Brown et al., 2001, Williams et al., 2002). The power and synchrony of these signals decreases following therapeutically effective dopamine replacement and deep brain stimulation (Marsden et al., 2001, Williams et al., 2002, Kühn et al., 2008). Consequently, cortico-subthalamic beta oscillations, and those in the wider basal ganglia network, have been proposed as a putative pathophysiological mechanism in PD (Williams et al., 2002, Eusebio and Brown, 2009).

Elucidating the mechanism by which pathological beta oscillations are generated and propagated through different parts of the network is crucial to advancing this hypothesis (Brown, 2007, Stein and Bar-Gad, 2012). Frontal/midline EEG signals (assumed to reflect the supplementary motor area) lead STN local field potentials (LFPs) with a time delay of 20–30 ms (Williams et al., 2002, Fogelson et al., 2006). In contrast, cortical beta oscillations recorded using ECoG electrodes over primary motor cortex led STN phase-locked spiking, revealed using spike trigged averages, by around 100 ms (Shimamoto et al., 2013). Due to differences in recording and analysis methods in these studies, it is not clear to what extent the dynamics of synchrony differ between single units and LFPs, and/or between different cortical areas.

During movement, cortico-subthalamic LFP beta desynchronisation correlates with motor performance (Kühn et al., 2004, Tan et al., 2015, Tan et al., 2016). Such task related changes in oscillation and synchronisation could reflect a role for these activities in healthy movement (Tan et al., 2016). In contrast, traditional models of basal ganglia function, based on recordings of single neurons in the STN and other BG nuclei, focus on widespread changes of the firing rate of individual neurons in coding movement parameters (Alexander and Crutcher, 1990, DeLong, 1990, Mink, 1996). It is unclear if, and how, such movement related changes in STN unit firing rate and pattern relate to beta synchronisation. Specifically, to what extent do individual STN neurons uncouple from cortical beta oscillations during movement-related firing rate changes? If STN neurons do not completely uncouple from cortical activity, does the angle of phase locking change in comparison to resting activity? Such questions have assumed greater significance due to the development of closed-loop deep brain stimulation, where the time delays between cortical oscillations and basal ganglia stimulation are an important determinant in the ability of these approaches to reduce motor symptoms (Rosin et al., 2011).

Here we demonstrate that the spikes of individual STN neurons are locked to cortical beta oscillations with constant time delays that differ between frontal/midline and lateral cortical areas. During periods of self-paced movement, many units only partially desynchronise from frontal and midline cortical beta oscillations and maintain the same phase locking dynamics that occur during rest. In addition, the temporal and spatial extent to which STN units are phase locked to midline cortical beta oscillations is highly correlated with the patients' akinetic/rigid symptoms. Together, these results show that the dynamics of beta synchronisation between STN neurons and premotor-cortical beta oscillations are highly stable and could contribute to motor impairment in PD.

## 2 Methods

All statistical values reported in the methods section are given as mean ± standard deviation (SD) unless noted otherwise.

### 2.1 Patient details and clinical scores

This study was conducted in agreement with the Code of Ethics of the World Medical Association (declaration of Helsinki, 1967) and was approved by the local ethics committee. All patients gave their written informed consent to participate in this study. We studied 12 patients (7 female, 5 male; age: 65 ± 6 years) suffering from advanced idiopathic PD (Hoehn & Yahr score: 3.5 ± 0.7, (Hoehn and Yahr, 1967) with a disease duration of 16 ± 5.8 years. Patients underwent bilateral microelectrode-guided implantation of DBS electrodes in the STN. Preoperatively, all patients had significant improvement of the Unified Parkinson's Disease Rating Scale motor section score (part III; (Fahn and Elton, 1987) following intake of levodopa (symptom improvement: 53% ± 14). Furthermore, all patients were classified as cognitively intact (based on their performance on the Mattis Dementia Rating Scale (Mattis, 1988) and fulfilled other inclusion criteria for STN-DBS, such as no structural alterations on magnetic resonance imaging (MRI), and no concomitant severe medical comorbidities. Further clinical details are summarised in Supplemental Table 1. The patient's motor UPDRS scores were assessed one week (5 ± 3 days) before the operation, both OFF and ON medication (motor score in Dopa-OFF condition: 39 ± 13 vs. Dopa-ON: 19 ± 9; *p* < 0.001, paired *t*-test).

### 2.2 Surgical procedures

Dopamine agonist treatment was stopped > 7 days before the operation, and surgery was performed after overnight withdrawal of anti-parkinsonian medication. Operations were performed under local anaesthesia. Details concerning the surgical procedure are reported elsewhere (Hamel et al., 2003, Moll et al., 2014). Briefly, a MRI-compatible Zamorano-Dujovny frame (Stryker Leibinger, Freiburg, Germany) was mounted on the patient's head and tightly secured with pins. Both gadolinium-enhanced volumetric T1 MRI and T2 weighted spin echo MRI sequences were acquired, and were fused with a computerized tomography scan using a commercially-available algorithm (iPlan, BrainLAB Inc., Westchester, IL, USA). Both commissures, the STN/nigra complex and vessels were delineated with high resolution. After determining a reference-line connecting the anterior and posterior commissure (AC-PC line), the STN was targeted 11–13 mm lateral to the midline, 1–3 mm inferior and 1–3 mm posterior to the mid-commissural point on both sides. The five parallel trajectories used for microelectrode recording (see below) avoided blood vessels, sulci, and ventricles. A burr hole (10 mm diameter) was fashioned anterior to the left and right coronal suture under local anaesthesia, the micromanipulator was mounted on the stereotactic frame and the appropriate target coordinates were adjusted. 10–15 min prior to the start of microelectrode recordings at thalamic level (typically 6–12 mm above the centre of the STN as delineated on MRI), systemic analgosedation with low dose remifentanil (0.01–0.1 μg/kg/min) was completely stopped. No sedatives or anesthetics were administered during the mapping procedure.

### 2.3 Microelectrode recordings

**Fig. 1:**
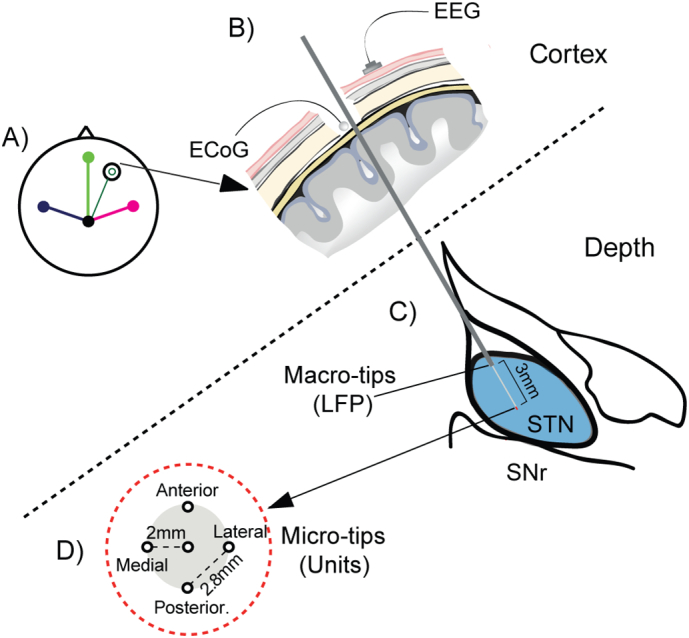
Intraoperative recordings of cortex and subthalamic nucleus. Schematic diagram of recording setup. (A) Cortical signals were recorded with 4 scalp EEG electrodes which were placed approximately in positions Fz, Cz, C3 and C4. (B) One ECoG electrode was placed directly on the dura under the burr hole adjacent to the inserted microelectrode (dark green circle). The burr hole was fashioned over dorsolateral prefrontal cortex. All signals were referenced or re-referenced against Cz. (C) LFPs were recorded from a macro-tip 3 mm above the micro-tip on the central, anterior and posterior electrodes. In some patients, an LFP was recorded from the micro-tip of the central electrode instead of the posterior macro-tip. (D) STN units were recorded from the micro-tip of one of 5 parallel microelectrodes in a cross arrangement.

**Fig. 2:**
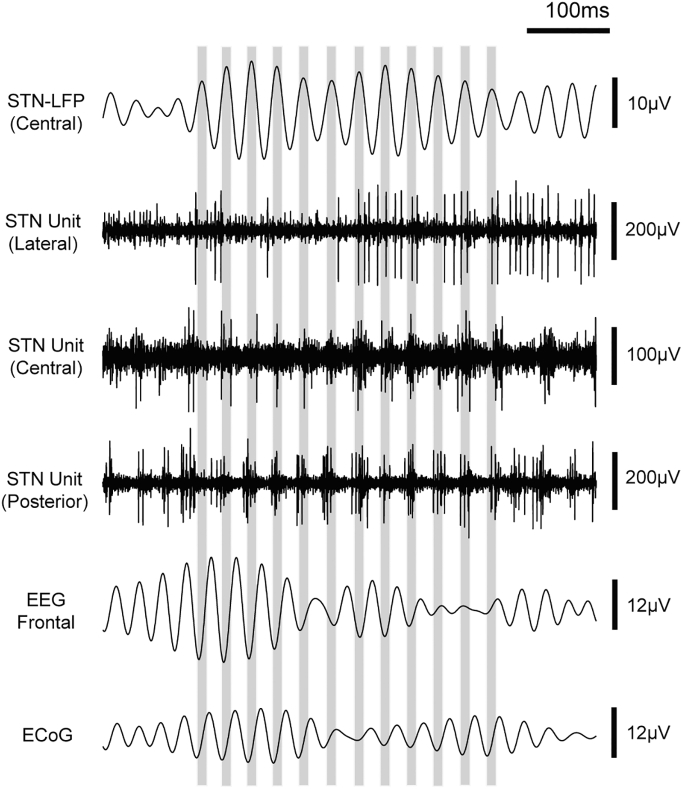
Representative recording of ongoing beta oscillations in STN units, LFP, EEG and ECoG. Top channel shows the beta frequency filtered (29–39 Hz) LFP recorded from the macro-tip of the central electrode, which was positioned within the STN. The grey bars show the peaks of the beta oscillations from this channel. The STN units recorded from the lateral microelectrode are both non-oscillatory and unsynchronised to the beta periods in the LFP. In contrast, the central and posterior units fire predominantly on the peak of the LFP oscillation. Beta oscillations in the midline EEG and ECoG recordings are also synchronised with the STN LFP beta oscillations at an inverse polarity to those in the STN LFP. Data was recorded in Patient 8. For LFP, EEG and ECoG signals, positive polarity is plotted upward.

Microelectrode recordings were performed along five parallel tracks arranged in a concentric array (MicroGuide, Alpha-Omega, Nazareth, Israel) (Fig. 1). Four outer platinum–iridium electrodes (impedance = 0.7 ± 0.2 (range, 0.2–1.25) megaOhm at 1000 Hz; FHC Inc., Bowdoinham, ME, USA) were separated by 2 mm from a central one, which was aimed at the theoretical target. Signals were amplified (× 20.000), bandpass-filtered (300–6000 Hz) and digitized (sampling rate: 24 kHz). Unit activities were identified as being from the STN using several previously described criteria (Sharott et al., 2014). An electrode was identified as having entered the STN following a clear increase in the size of the background activity (Moran et al., 2006). STN units were distinguished by their tonic irregular, oscillatory or bursty discharge pattern (Fig. 2), which was clearly different from slower bursting and single-spiking of neurons in the overlying thalamus/zona incerta and from the high frequency regular spiking of substantia nigra pars reticulata neurons found ventrally.

### 2.4 Cortical EEG and ECoG signals

In parallel with the deep brain signals, a 32 channel system (AlphaLab, Alpha Omega Inc., Nazareth, Israel) was used to amplify and record electroencephalographic (EEG, amplification × 4.000; bandpass, 0–400 Hz) and electromyographic (EMG; amplification × 2.000; bandpass, 5–1000 Hz) signals at a sampling rate of 3005 Hz during the microrecording and stimulation periods (Moll et al., 2015). EEG was recorded from 4 scalp electrodes (Ag/AgCl cup electrodes filled with conductive gel; Nicolet Biomedical, Madison, WI, USA) placed approximately at positions Fz, Cz, C3 and C4 according to the international 10–20 system for the placement of EEG electrodes, with the left earlobe as a common reference. In 7 patients, we additionally recorded an electrocorticogram (ECoG) from the dura above the craniotomy, approximately over the dorsolateral prefrontal cortex and referenced to the Cz channel (Fig. 1A–B). All EEG channels were also re-referenced to the Cz electrode to give three bipolar EEG channels which will be referred to from here on as midline (for the Fz/Cz derivation), and contralateral or ipsilateral (for the Cz to C3/C4 derivation in respect to the side of the depth micro- or macroelectrode being analysed). In one case, the Fz position was not recorded, and has therefore only been included in analyses of ECoG.

### 2.5 Data selection and processing

All data were collected as part of our routine neuronavigation process which aims to precisely identify the surgical target (STN). Patients were only included in this study if a minimum of 10 STN unit recordings, each lasting > 45 s, were recorded from one or both hemispheres (Sharott et al., 2014). It is important to note that recordings were only included if they were made during sustained periods where patients were awake and highly co-operative. In total, 269 STN unit recordings were used in this study. In some recordings (96/269 units recorded in 9/12 patients), patients were engaged in a simple, brief movement task (flexion/extension of wrist or ankle) as part of the routine mapping procedure.

Spike detection was performed offline using a voltage threshold method, the threshold value of which was set sufficiently high relative to the noise level to avoid false-positives (typically, threshold values > 4 SD of the background level were used). When possible, single unit activities (SUAs) were then separated by manual cluster selection in 3D feature space on the basis of several waveform parameters including principal components, signal energy, peak time and the presence of a central trough in the autocorrelogram (Offline-Sorter, Plexon Inc., Dallas, TX, USA). Over half of the unit activities were characterized as SUAs (*n* = 184). Given the high density of neurons in the STN, we chose to be conservative in spike sorting and in the classification of single units. Many recordings classified as multiunit activities (MUAs) may, therefore, have comprised mostly of one or two STN units and were clearly distinct from background spiking activity utilised in some previous studies (Moran et al., 2008, Zaidel et al., 2010). In all cases, the initial 5–15 s of the unit recording was discarded as were any portions of injury discharge at the end of the recording.

### 2.6 Signal processing

The firing rate of each neuron was calculated in the most stable part of the spike train. STN units are autonomously active (Do and Bean, 2003) and their tonic firing does not generally fall below 10 spikes/s in well isolated recordings (Magill et al., 2000, Mallet et al., 2008b). The firing rate of each neuron was calculated using the longest part of the spike train where the firing frequency was above 10 spikes/s in every 2 s bins for > 30 s. By doing this, we attempted to ensure that mean firing rate was not affected by recording instability. The mean rate of single units was 33.7 spikes/s, in agreement with previous studies with well-isolated units in parkinsonian animals and humans (Bergman et al., 1994, Weinberger et al., 2006, Mallet et al., 2008b, Sharott et al., 2014).

Spectral parameters for both time-series and point-processes were evaluated using fast Fourier transform (FFT) as described in Halliday et al. (1995). A Hanning window filter was used for all spectral analyses and spectra were estimated by averaging across these discrete sections (Halliday et al., 1995). Spectral analyses and related correlations were computed with a combined population of single- and multi-units and were calculated using the MATLAB toolbox Neurospec (www.neurospec.org). Coherence and phase analyses were used to evaluate the strength and temporal delay of coupling between the EEG/ECoG and STN unit/LFP channels respectively. The cross- and autospectra from two time series can be combined to give the coherence: a measure of the degree to which one can linearly predict change in one signal given a change in another signal (Brillinger, 1981; Halliday et al., 1995; Rosenberg et al., 1989). Being a normalised measure, coherence is bounded from 0 to 1, with a value of 0 indicating non-linearly related signals and a value of 1 signifying two linearly identical signals. Significance was evaluated using confidence limits based on the number of segments used and was independent of frequency (Halliday et al., 1995). The variance of the coherence at each frequency was normalised by transforming its square root using the Fisher transform (Brown et al., 2001, Sharott et al., 2005).

When coherence between time series/point processes is significant, the phase spectra, defined as the argument of the cross-spectrum, can be used to calculate the time delay between two time series over a given frequency range (Halliday et al., 1995, Fogelson et al., 2006, Magill et al., 2006). Using a series of contiguous frequency bins, rather than a single frequency point to evaluate the phase delay is advantageous, as the latter is ambiguous (Halliday et al., 1995, Fogelson et al., 2006). Over frequency ranges with significant coherence (> 6 contiguous bins over the 95% confidence interval), the time lag/lead between two signals was calculated from the slope of the phase estimate, having fitted a line by linear regression to establish the slope as significantly different from zero (Halliday et al., 1995, Fogelson et al., 2006). A significant positive or negative gradient indicates a lag or lead by the input channel respectively relative to the reference channel, at a latency given by the slope of the gradient divided by 2π (Halliday et al., 1995). The time delay was calculated when the phase gradient (corresponding to the significantly coherence bins) had *p*-value of < 0.05 and an R^2^ value of > 0.5.

### 2.7 Phase locking analysis

To further investigate how the activity of STN units varied in time with respect to ongoing cortical activity, we analysed the instantaneous phase relationships between STN spike times and cortical oscillations in narrow frequency bands. EEG, ECoG and LFP signals were first filtered, using a neutral-phase bandpass filter (Butterworth filter, 2nd or 3rd order) in 13, 5 Hz wide, frequency bands from 5 to 40 Hz with a 2.5 Hz overlap. Subsequently, the instantaneous phase and power of the ECoG in these frequency bands were separately calculated from the analytic signal obtained via the Hilbert transform (Lachaux et al., 1999). In this formalism, peaks in the oscillation correspond to a phase of 0° and troughs to a phase of 180°. Circular phase plots and circular statistical measures were calculated using the instantaneous phase values for each spike. Descriptive and inferential circular statistics were then calculated using the CircStat toolbox (Berens, 2009) for MATLAB. Units were first tested for significantly phase-locked firing (defined as having *p* < 0.05 in Rayleigh's uniformity test). The null hypothesis for Rayleigh's test was that the spike data were distributed in a uniform manner. For each of the neurons that fulfilled these criteria, the mean phase angle was calculated. The mean resultant vector length (referred to hereafter as simply ‘vector length’) of the phase distribution, bound between zero and one (the closer to one, the more concentrated the angles), was used to quantify the level of phase locking around the mean angle for individual neurons (computed using the angles of each spike) and for populations of neurons (computed using the mean angle for each neuron).

**Supplemental Fig. 1:**
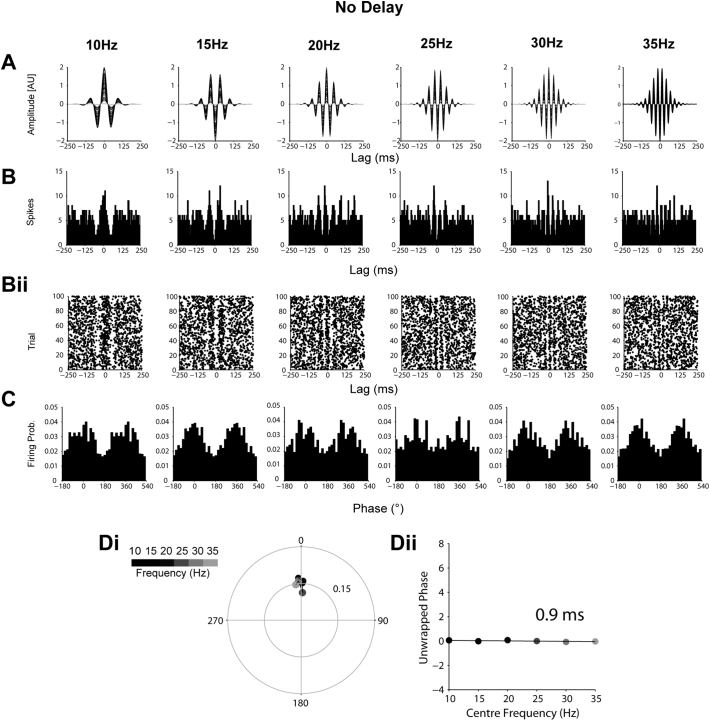
Simulated data showing the derivation of zero delay from modulation of firing probability by transient oscillations. (A) To represent cortical beta oscillations, sine waves (1000 points) from 10 to 35 Hz were multiplied with a 1000 different Gaussian functions of variable, but proportional, peak height and width. (Bi) STN unit activity in each trial was represented by a Poisson point process, whereby at each sampling point there was a 0.032 probability of a “spike”, to give a “firing rate” of 32 spikes per second. In each trial, this baseline probability of firing was weighted by multiplying with the matching point of a randomly selected beta “oscillation” from A. (Bii) This weighting of the probability of firing leads to peaks and troughs of the sum of all the spike trials being aligned to those of the oscillations in A. (C) Phase histograms derived from instantaneous phases of the oscillations in A at the times of the spikes in B. At all frequencies, the maximal firing is at the peak of the oscillation. (D) The mean phase angle (position on circle) and vector length (distance from centre) of phase locking for each frequency, given by the colour scale. Note that both mean angle and vector length are constant across all frequencies. (Dii) Phase values from Di unwrapped to show their progression on a linear scale. The negligible delay derived from this phase progression (0.9 ms, inset) reflects that the two signals were synchronised with zero lag.

**Supplemental Fig. 2:**
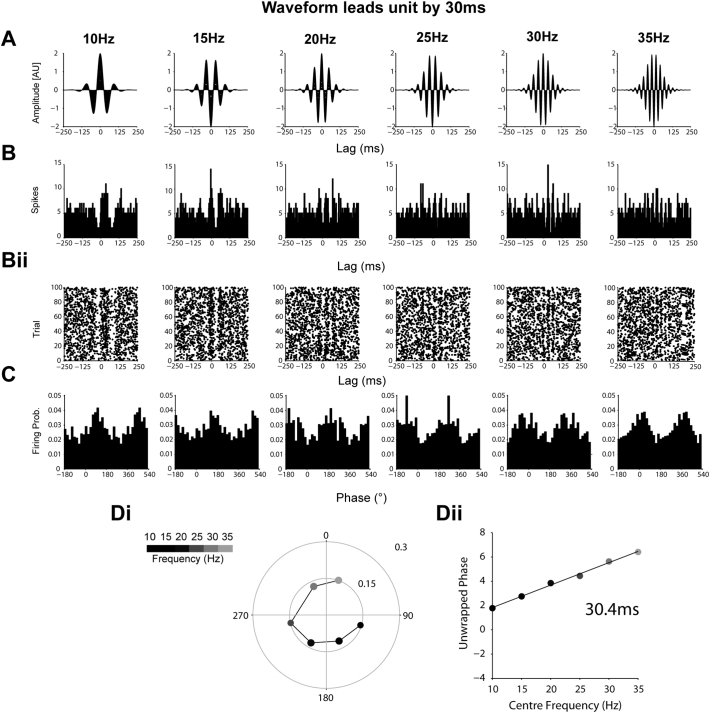
Simulated data showing the derivation of 30 ms lead from modulation of firing probability by transient oscillations with a fixed time delay. (A–B) As in Supp. Fig. 1, but with a fixed 30 ms delay between the probability weighting of the spikes by the oscillations in A. (C) The maximum angle of firing now phase advances as frequency increases. (Di) The circular representation demonstrates that the mean angle of locking moves 270° between 10 and 35 Hz. (Dii) The 30 ms delay can be derived from this phase progression on the linear scale.

**Supplemental Fig. 3:**
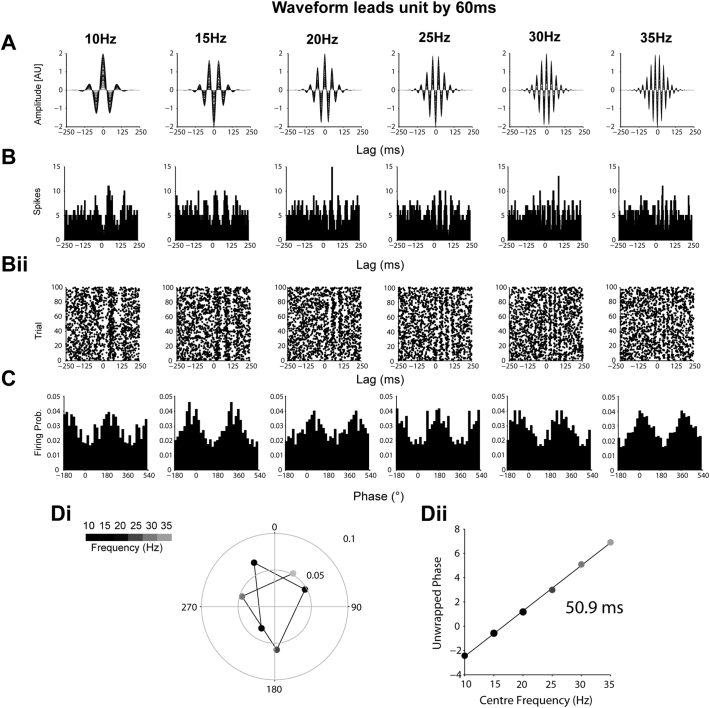
Simulated data showing the derivation of 60 ms lead from modulation of firing probability by transient oscillations with a fixed time delay. (A–B) As in Supp. Fig. 1, but with a fixed 60 ms delay between the probability weighting of the spikes in by the oscillations in A at all frequencies. (C) The maximum angle of firing now phase advances as frequency increases. (Di) The circular representation demonstrates that the mean angle of locking move over a cycle between 10 and 35 Hz. (Dii) The 60 ms delay can be derived from this phase progression on the linear scale.

**Supplemental Fig. 4:**
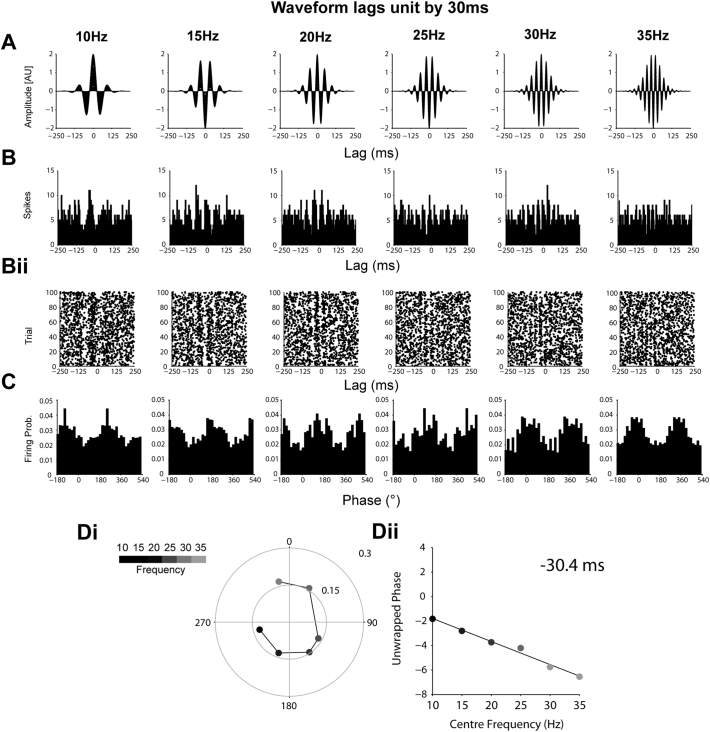
Simulated data showing the derivation of 30 ms lag from modulation of firing probability by transient oscillations with a fixed time delay. (A–B) As in Supp. Fig. 1, but with a fixed − 30 ms delay between the probability weighting of the spikes in by the oscillations in A at all frequencies. (C) The maximum angle of firing now phase regresses as frequency increases. (Di) The circular representation demonstrates that the mean angle of locking moves around 270°, but it the opposite direction to that in Supp. Fig. 2. (Dii) The − 300 ms delay can be derived from this phase regression on the linear scale.

Time delays were calculated from instantaneous phase-based analysis in a similar way to that described for spectral analysis. The mean phase of units that were significantly locked (Rayleigh test, *p* < 0.05) in each band were averaged to give a grand average phase for each frequency band. A significant positive or negative gradient indicates a lag or lead by the EEG/LFP/ECoG channel respectively relative to the reference channel, at a latency given by the slope of the gradient divided by 2π. Under the assumption that the oscillation modulates the probability of firing of the unit with a fixed delay across different frequencies, we used simulated data (Supplemental Fig. 1, Supplemental Fig. 2, Supplemental Fig. 3, Supplemental Fig. 4) to show that this method allows the delay to be calculated with high accuracy. STN units often lock to cortical signals within a narrow frequency range (Shimamoto et al., 2013), making spectral analysis of the time delay between these signals suboptimal. Calculating the time delay in this manner allowed us to more easily pool the circular means of different neurons that locked to a narrow part of the wider beta frequency range. To test the consistency of this time lag for a given channel, the analysis was repeated 250 times using randomly selected mean angles from each frequency band. The phase delay was then calculated from this random sample both in the correct order and in a shuffled order. Histograms of the resulting time delays were then calculated for both samples and the histogram of real angles was expressed as a z-score of histogram of the shuffled angles (i.e. how many standard deviations from the shuffled histogram was the real histogram). This process was repeated 250 times and mean and 95% confidence levels of the z-score calculated. A significant peak in the z-score (> 2) for a given lag therefore indicates that the lag occurs more than would be expected by chance. This analysis will not result in a peak at zero when there is consistently no delay between the two signals, as both the shuffled and real data will not produce gradients, leading to low z-scores at all delays.

### 2.8 Analysis of rest and movement periods

Movement periods, consisting of continuous flexion/extension of the wrist contralateral to the recorded STN, were isolated using EMG recordings from the flexor and/or extensor muscles. All movement periods were examined by eye, and those with 2 or more sustained rest (11.2 ± 6.5 s) and movement periods (17.8 ± 8.2 s), with a minimum of 4 s, were defined using a threshold set by visual inspection. 42 STN units from 5 patients, of which 30 were SUAs, where recorded during such rest/movement periods. For each unit, the firing rate was calculated across movement and rest periods and converted to percentage change during movement. As we were interested in the relationship between firing rate and oscillation, for this analysis we normalised the mean resultant vector length against that of 100 ISI-shuffled surrogate spike trains to ensure the number of spikes could not confound the phase locking measure (Rivlin-Etzion et al., 2006, Sharott et al., 2009, Sharott et al., 2014). The vector length was therefore expressed as a z-score (the number of standard deviations of the vector length of the real spike train compared to this surrogate distribution). In order to compare with the % firing rate change, the phase-locking z-score for movement was subtracted from the rest z-score to give a single desynchronisation value. To evaluate the power of the cortical signals during these rest and movement periods, the amplitude envelope from the Hilbert-transformed beta-filtered data (at the frequency at which there was a phase-locked unit) for the entire rest and movement period was normalised to a z-score. The mean amplitude of the rest and movement periods was then calculated from this normalised data, allowing comparison of signal amplitude in rest and movement periods across recordings from different patients.

### 2.9 Correlation of phase locking and clinical scores

Correlations between physiological parameters and clinical UPDRS sub-scores were calculated as described in Sharott et al., 2014. The percentage of STN units that were significantly locked to the midline EEG (recorded in all but one patient, *n* = 11) and the mean resultant vector length were correlated with the combined rigidity (subscore 22) and akinesia/bradykinesia (subscores 23–26 and 31) scores OFF medication across patients. For the percentage phase locked units, if a unit was significantly locked (Rayleigh test, *p* < 0.05) to any of the sub-bands within the low (12–24) or high (25–35) beta range (e.g. 10–15 Hz within the low beta range), that unit was counted as significantly locked for the wider range. For the mean resultant vector length, the maximum vector length for each unit was calculated for each frequency band, and these were averaged across all the units recorded in that patient. A bivariate regression model was used to calculate the Pearson correlation coefficient (R^2^) and associated *p*-value to evaluate the strength, significance and the best linear fit of the correlation. All Pearson correlation values had a Cook's distance of > 1 suggesting that there were no outlying points (Cook, 1977).

## 3 Results

### 3.1 Cortico-subthalamic recordings in PD patients

**Supplemental Fig. 5:**
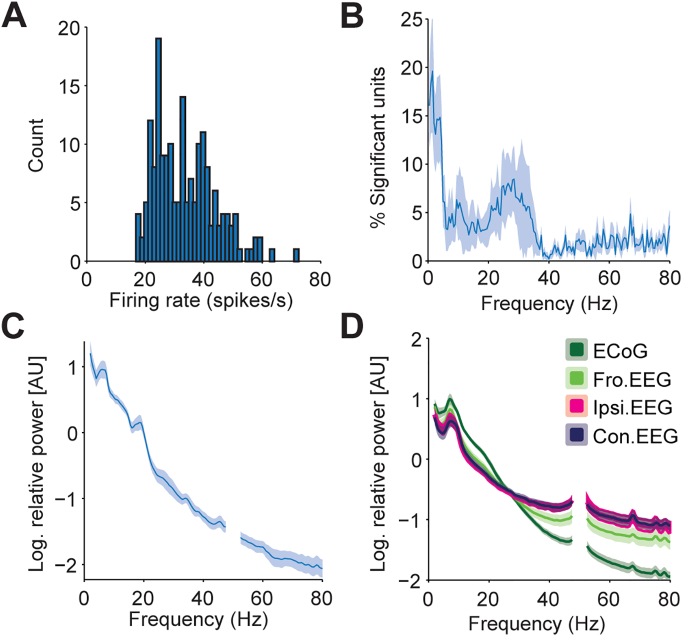
Cortical and subthalamic signal properties. (A) Histogram of firing rates across all single units recorded in this study (*n* = 184). (B) Histogram of percentage of units with significant power from 1 to 80 Hz across all patients (*n* = 12). (C) Mean relative spectral power of STN LFPs across patients where those signals were recorded (*n* = 11). (D) Mean relative spectral power of ECoG (*n* = 7), midline EEG (*n* = 11), ipsilateral EEG (*n* = 10) and contralateral EEG (*n* = 10) across all patients where those signals were recorded. Shaded areas show ± SEM across patients.

Our aim was to define the dynamics of beta frequency synchronisation between STN neurons and different cortical areas in patients with PD. To this end, we recorded surface EEG from 3 positions on the scalp using bipolar derivations with a central electrode, resulting in one midline and two lateral channels (defined as ipsilateral and contralateral to the coincident STN recording, Fig. 1A). In addition, in 7 patients we were able to record ECoG signals through a silver-ball inserted into the burr hole, approximately over the prefrontal cortex (Fig. 1B). STN-LFPs could be recorded from either macro-tips or the central micro-tip in 11 patients (Fig. 1C). STN single and multiunit activities were recorded from multiple tungsten microelectrodes (Fig. 1D). Synchronisation between STN unit activities and beta oscillations in the LFP and cortical activities could often be observed by eye (Fig. 2). The mean (33.67 spikes/s) firing rate of SUAs (Supplemental Fig. 5A) was in good agreement with well-isolated units in parkinsonian animals (Mallet et al., 2008b) and patients (Sharott et al., 2014). As reported previously (Weinberger et al., 2006, Sharott et al., 2014), the power spectra of subthalamic neuron spike trains and STN LFPs displayed peaks in the sub-beta and beta frequency ranges (Supplemental Fig. 5B, C). The mean power spectra of the ECoG and EEG only had a prominent peak in the sub-beta, but not beta range (Supplemental Fig. 5D). ECoG and midline-EEG channels appeared to have higher beta and lower gamma power than the lateral channels, but this was not statistically significant (Kruskal-Wallis ANOVA, *p* < 0.05).

### 3.2 LFPs underestimate subthalamic synchronisation with lateral cortical areas

**Fig. 3:**
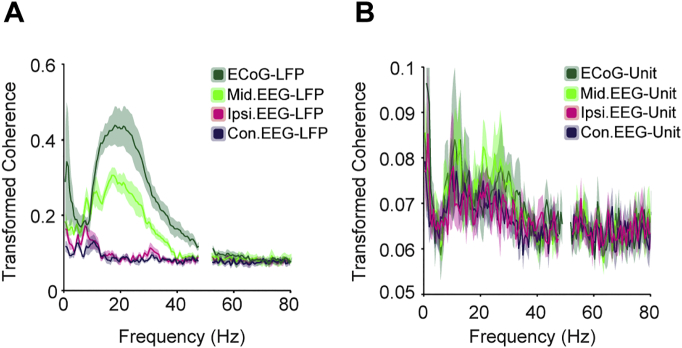
Spectral relationships between cortex, STN unit and LFP recordings. (A) Mean coherence between the cortical channels and STN LFP across all patients where those signals were recorded (ECoG: *n* = 7; EEG channels: *n* = 9). Coherence across the whole beta range (12–35 Hz) was significantly larger for the ECoG and midline-EEG pairs than for ipsilateral and contralateral pairs (Fig. 4A, Kruskal-Wallis, *p* = 0.000008, post-hoc Dunn's tests). (B) Mean coherence between the cortical channels and STN units coherence across all patients where those signals were recorded (ECoG: *n* = 7; EEG channels: *n* = 9). There were no significant differences in the coherence between midline and lateral EEG-STN-unit pairs in any frequency band. All coherence values were Fisher-transformed.

Spectral analysis of post-operative EEG and STN LFP recordings suggests that STN is more highly synchronised with frontal/mesial, than lateral channels (Marsden et al., 2001, Williams et al., 2002, Fogelson et al., 2006). Our analysis of the coherence between EEG and STN LFPs recorded intraoperatively produced very similar results (Fig. 3A). The frontal ECoG was even more coherent with the STN than the midline EEG, but with a similar frequency profile (Fig. 3A). Across the whole beta range (12–35 Hz), coherence was significantly greater for the ECoG and midline-EEG pairs than for ipsilateral and contralateral pairs (Fig. 3A, Kruskal-Wallis, *p* = 0.000008, post-hoc Dunn's tests). If STN LFPs are a simple reflection of synchronisation of the underlying neuronal activity, a similar topographical relationship would be expected for the single STN units, but this was not the case (Fig. 3B). There were no significant differences in the coherence between midline and lateral EEG-STN-unit pairs in the beta range Kruskal-Wallis, *p* > 0.05).

**Supplemental Fig. 6:**
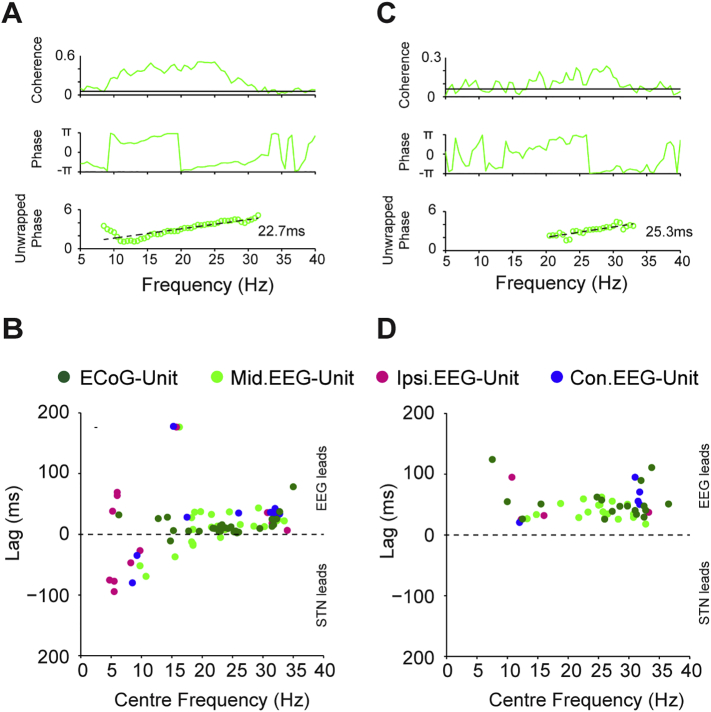
Phase spectra-based estimation of corticosubthalamic time delays for STN LFPs and units. (A) Example of time delay derived from the coherence (top) and phase spectra (middle) of a single midline-EEG/STN LFP pair. The time delay is derived from the gradient of the unwrapped phase spectrum (bottom) where there is significant coherence. (B) Time delays derived from the phase spectra of EEG/ECoG and STN-LFPs pairs where the gradient of the phase spectra was significantly different to zero and there was a significant peak in the corresponding coherence spectrum. Colour code as in panels A/B. Positive lags indicate EEG leads and negative lags indicate STN-LFP leads. Over all midline EEG-STN LFP (*n* = 28) or ECoG-STN LFP (*n* = 32) pairs where phase could be calculated, the cortical signals led the STN LFP by a mean of 13.4 ± 6 ms in the low-beta (*n* = 33, 12–24 Hz) and 19.4 ms in the high-beta (*n* = 23, 24–35 Hz) ranges, respectively. (C–D) As in panels A/B, but for EEG/ECoG and STN unit pairs. The time delay between cortex and STN units was derived from gradient in the phase spectra of significant coherent STN unit - midline EEG/ECoG pairs (EEG: *n* = 19, ECoG: *n* = 19). As with the STN LFP, the EEG and ECoG consistently led the STN units in beta range, but at longer latencies as compared to the STN-LFP, in the low-beta (*n* = 8, 39 ± 5 ms) and high-beta (*n* = 26, 45 ± 4 ms) ranges.

Analysis of phase spectra-based time delays concurred with previous work suggesting that frontal cortical beta oscillations “lead” those in the STN LFP (Williams et al., 2002, Fogelson et al., 2006, Lalo et al., 2008). Over all EEG-STN LFP (*n* = 28) or ECoG-STN LFP (*n* = 35) pairs where phase could be calculated, the cortical signals led the STN LFP by a mean of 17.6 ± 7 ms and 19.4 ± 2 ms in the low-beta (*n* = 33, 14–24 Hz) and high-beta (*n* = 23, 24–35 Hz) ranges, respectively (Supplemental Fig. 6A, B). The time delay between cortex and STN units was derived from gradient of the phase spectra of significantly coherent STN unit - midline EEG and frontal ECoG pairs (EEG: *n* = 56, ECoG: *n* = 33, Supplemental Fig. 6C, D). As with the STN LFP, the EEG and ECoG consistently led the STN units in beta range, but at slightly longer latencies, in the low-beta (*n* = 6, 40 ± 5 ms) and high-beta (*n* = 28, 43.2 ± 4 ms) ranges (Supplemental Fig. 6D). In contrast to STN LFP, there were few significant EEG-unit pairs in the sub-beta range.

In summary, the STN LFP only partially predicted the temporal and spatial parameters of STN unit synchronisation to cortical oscillations.

### 3.3 Frequency dependence of phase locking reveals topographically specific cortico-subthalamic time delays

**Supplemental Fig. 7:**
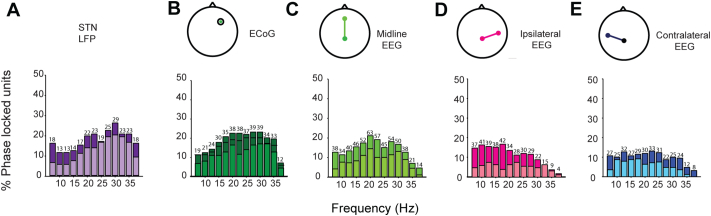
Percentage of STN units locked to different cortical channels across the beta range. (A–E) Histograms of the percentage of all units significantly phase locked to the STN LFP (A), ECoG (B), midline EEG (C), ipsilateral EEG (D) and contralateral EEG (E) in overlapping 5 Hz frequency bands filtered from 7.5 to 37.5 Hz. Dark colors show the proportion of significant neurons locked at *p* < 0.05. The n number of significant neurons for each frequency at this significance level, which were included in the analysis in Fig. 6, is shown above each bar in each plot. Light colors show the percentage of significant neurons following Bonferroni correction for testing across the 5 different signals (*p* < 0.01).

Overall, comparison of STN LFP and unit coherence with the cortical signals suggested that LFPs may underestimate the coupling of lateral cortical channels. In addition, peaks in coherence between motor cortical oscillations and STN units are often sharp (Shimamoto et al., 2013), rendering spectral estimates for defining the synchronisation parameters of STN units suboptimal. Thus, we further analysed the coupling of STN units to cortical beta oscillations by examining the phase locking of spike trains to 5 Hz bands within the broader beta band for STN LFPs and each cortical signal. Up to 20 to 25% of STN units recorded were phase locked to beta oscillations the STN LFP using this method, depending on the significance level used (Rayleigh Test, *p* < 0.01/0.05; Supplemental Fig. 7A). In agreement with coherence analysis (Fig. 3B), the proportion of STN units that were significantly phase-locked at beta frequencies varied relatively little between cortical channels (Rayleigh Test, *p* < 0.05; Supplemental Fig. 7B–E). Having established that pool of STN neurons locked to oscillations at different frequencies across the beta range, we used the mean phase angle of locking to these narrow beta bands to recalculate the time delays between STN units and LFP, ECoG and EEG signals. This technique allowed us to pool neurons that synchronised to narrow frequency bands and to derive time delays from this pooled data (Supplemental Fig. 1, Supplemental Fig. 2, Supplemental Fig. 3, Supplemental Fig. 4).

**Fig. 4:**
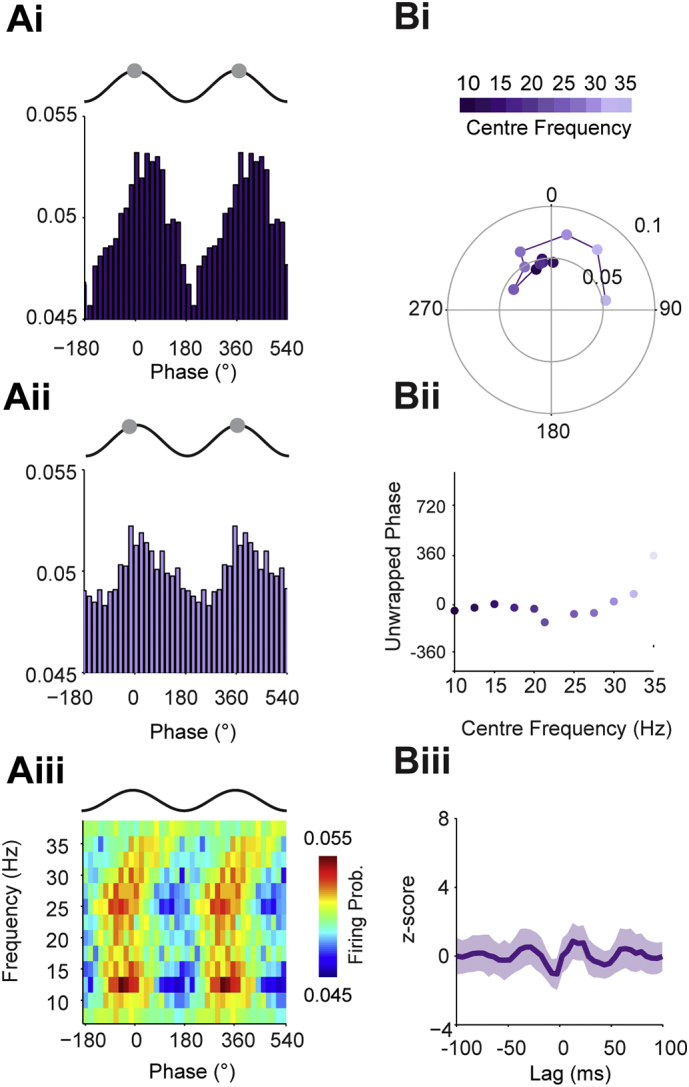
STN units fire at zero time delay with respect to the STN LFP. (Ai–ii) Mean phase histograms of all STN units significantly phase locked (Rayleigh test, *p* < 0.05) to the STN-LFP from the left hemisphere of Patient 11 at low beta frequencies (Ai, 10–20 Hz centre frequencies) and high beta frequencies (Aii, 22.5–35 Hz centre frequencies). For clarity, two cortical slow oscillation cycles are shown. Grey circles show the maximal phase of firing, which is at the peak of the oscillation cycle for both frequencies. (Aiii) Mean phase histograms of significantly locked units across all patients using overlapping 5 Hz frequency bands from 7.5 to 37.5 Hz. Firing probability is now shown on the colour scale. (Bi) Mean angle (position on circle) and vector length (distance from centre) of phase locking for all units significantly phase locked to the STN LFP pairs at frequencies from 10 to 35 Hz, as indicated by colour scale above. The preferred phase of the units was near the peak of the STN LFP oscillation, irrespective of frequency. (Bii) Phase values from Bi unwrapped to show their progression on a linear scale. The flat line confirms that there is no delay between the two activities. (Biii) Mean z-score of resampled delays of the STN unit - STN LFP pairs suggests there is no significant delay between STN units and LFPs.

**Fig. 5:**
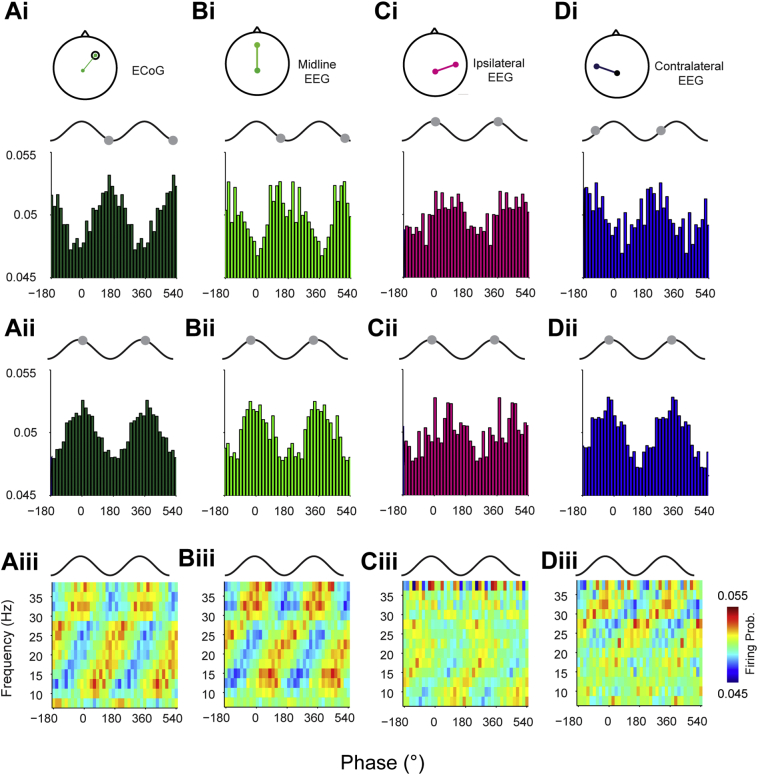
Phase locking of STN units to cortical beta oscillations is topographically and frequency specific. (Ai–ii) Mean phase histogram of all units significantly phase locked (Rayleigh test, *p* < 0.05) to the STN-LFP from the left hemisphere of Patient 11 at low beta frequencies (Ai, 10–20 Hz centre frequencies) and high beta frequencies (Aii, 22.5–35 Hz centre frequencies). For clarity, two cortical slow oscillation cycles are shown. Grey circles show the maximal phase of firing in each case. (Aiii) Mean phase histograms of significantly locked units across all patients using overlapping 5 Hz frequency bands from 7.5 to 37.5 Hz. Firing probability is now shown on the colour scale. (B–D) As in A, but for the midline EEG (B), ipsilateral (to the STN) EEG (C) and contralateral EEG (D).

Firstly, we analysed the relationship between STN units and the STN LFPs, where there should be little or no delay between the two signals. As expected, phase of maximal firing of STN units in relation to the STN LFP was constant, on the peak of the oscillation, across the whole beta range. This was evident at the level of single subjects (Fig. 4A) and across the whole data set (Fig. 4B). The mean phase distribution across the frequency ranges was flat, suggesting negligible time delay between the STN units and LFPs (Fig. 4Bii, and see Supp. Fig. 1Dii for comparison). Recomputing this delay for randomly selected, phase locked LFP-unit pairs across the beta frequency range led to a distribution of delays with no clear peaks (Fig. 4Biii), suggesting that there was no consistent delay across the sample of LFP-unit pairs (note that zero delay will not result as a peak as the real and shuffled data both give flat distributions). In contrast, phase histograms for the ECoG and midline EEG channels were approximately antiphase between low and high beta frequencies (Fig. 5Ai–ii, Bi–ii). This was the result of a linear shift in the phase of maximal firing as the centre beta frequency increased (Fig. 5Aiii, Biii). For the ipsilateral EEG, the phase histograms low and high frequencies looked similar, with maximal firing on the descending phase of the oscillation (Fig. 5C). Maximal firing to the contralateral EEG was shifted by around a quarter cycle between low and high beta frequencies (Fig. 5Di–ii). Plotting the firing probability across all frequencies revealed that this was caused by the maximal firing phase moving linearly over two cycles across the entire beta range (Fig. 5Diii), suggesting a longer lag between the contralateral EEG and STN units than for the frontal and midline channels.

**Fig. 6:**
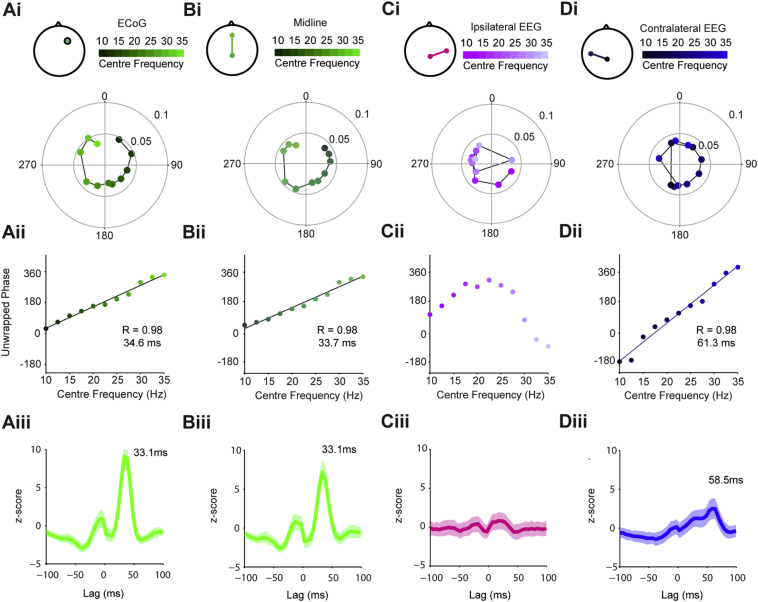
Time delays between cortical beta oscillations and STN units are topographically specific. (Ai) Circular plots showing mean angles (position on circle) and vector lengths (distance from centre) of STN units that were significantly phase locked to the ECoG at frequencies from 10 to 35 Hz (centre frequency of overlapping 5 Hz bands as indicated by colour scale above). (Aii) Phase values from Ai unwrapped to show their progression on a linear scale. There was a significant, linear relationship between frequency and phase. The time delay derived from the gradient of the linear fit was 34.6 ms. (Aiii) To examine the consistency of this relationship, the phase delay was calculated using a bootstrapping method and a z-score calculated against shuffled surrogates. This analysis showed a clear, significant peak at virtually the same delay as the mean data (Aii). (Bi–Biii) As in A, but for midline EEG-STN unit pairs. The results in each case are similar to those for the ECoG. (Ci–iii) The same analysis of ipsilateral-EEG shows that STN units fire on the ascending phase of the cortical oscillations across most of the beta range (i–ii). Bootstrapping analysis (iii) confirms that there is no consistent delay between these signals. (Di–iii) In contrast, the firing of STN units phase-advances with frequency in relation to the contralateral EEG (i). This relationship is significantly linear, but the phase delay derived from its slope (inset) is approximately twice that of the ECoG and midline EEG (ii). Although consistency of this delay is not as strong as for the frontal channels (iii), it is significant (i.e. z-score > 2). (The number of neurons included for each frequency band for each cortical signal is shown in Supplemental Fig. 6). Time delays between cortical beta oscillations and STN units are topographically specific. (Ai) Circular plots showing mean angles (position on circle) and vector lengths (distance from centre) of STN units that were significantly phase locked to the ECoG at frequencies from 10 to 35 Hz (centre frequency of overlapping 5 Hz bands as indicated by colour scale above). (Aii) Phase values from Ai unwrapped to show their progression on a linear scale. There was a significant, linear relationship between frequency and phase. The time delay derived from the gradient of the linear fit was 34.6 ms. (Aiii) To examine the consistency of this relationship, the phase delay was calculated using a bootstrapping method and a z-score calculated against shuffled surrogates. This analysis showed a clear, significant peak at virtually the same delay as the mean data (Aii). (Bi–Biii) As in A, but for midline EEG-STN unit pairs. The results in each case are similar to those for the ECoG. (Ci–iii) The same analysis of ipsilateral-EEG shows that STN units fire on the ascending phase of the cortical oscillations across most of the beta range (i–ii). Bootstrapping analysis (iii) confirms that there is no consistent delay between these signals. (Di–iii) In contrast, the firing of STN units phase-advances with frequency in relation to the contralateral EEG (i). This relationship is significantly linear, but the phase delay derived from its slope (inset) is approximately twice that of the ECoG and midline EEG (ii). Although consistency of this delay is not as strong as for the frontal channels (iii), it is significant (i.e. z-score > 2). (The number of neurons included for each frequency band for each cortical signal is shown in Supplemental Fig. 6).

We utilised the significantly phase locked units across the data set to derive time delays from these phase/frequency relationships for each cortical channel (Fig. 6). The number of neurons included in the analysis for each frequency band for each cortical signal is shown in Supplemental Fig. 7. For the frontal ECoG and midline EEG channels, the preferred angle of firing increased linearly with the cortical frequency, moving from the descending to ascending phase of the oscillation (Fig. 6Ai, Bi). The gradients of the slope of the unwrapped phase of this pooled data translated to a time delays of 34.6 ms and 33.7 ms for the ECoG and midline EEG, respectively (Fig. 6Aii, Bii). Using bootstrapping statistics, we then tested the consistency of these delays across the sample of significantly locked neurons. This analysis showed a clear peak at 33 ms for both channels (Fig. 6Aiii, Biii). In contrast, the mean preferred angle for the ipsilateral channel showed no such frequency/phase relationship (Fig. 6C), with the mean phase angle centred around 270° for most frequencies (Fig. 6Ci), resulting in no significant delay (Fig. 6Cii–iii). For the contralateral EEG, the mean values also had a linear relationship between mean phase angle and frequency, but with a steeper slope than the frontal channels, translating to a delay of 60.13 ms (Fig. 6Di–ii). This could be seen in the group analysis (peak at 58 ms), albeit more weakly than for the frontal channels (Fig. 6Diii).

In summary, phase locking-based analysis enabled the detection of significant beta synchronisation between frontal, midline and lateral areas. STN units lagged frontal and midline cortical channels with the most consistent time delays. Although more weakly locked, beta oscillations in the contralateral cortex preceded those in STN neurons by approximately twice the delay of the frontal and midline channels.

### 3.4 Cortico-subthalamic beta dynamics are maintained during movement desynchronisation

**Fig. 7:**
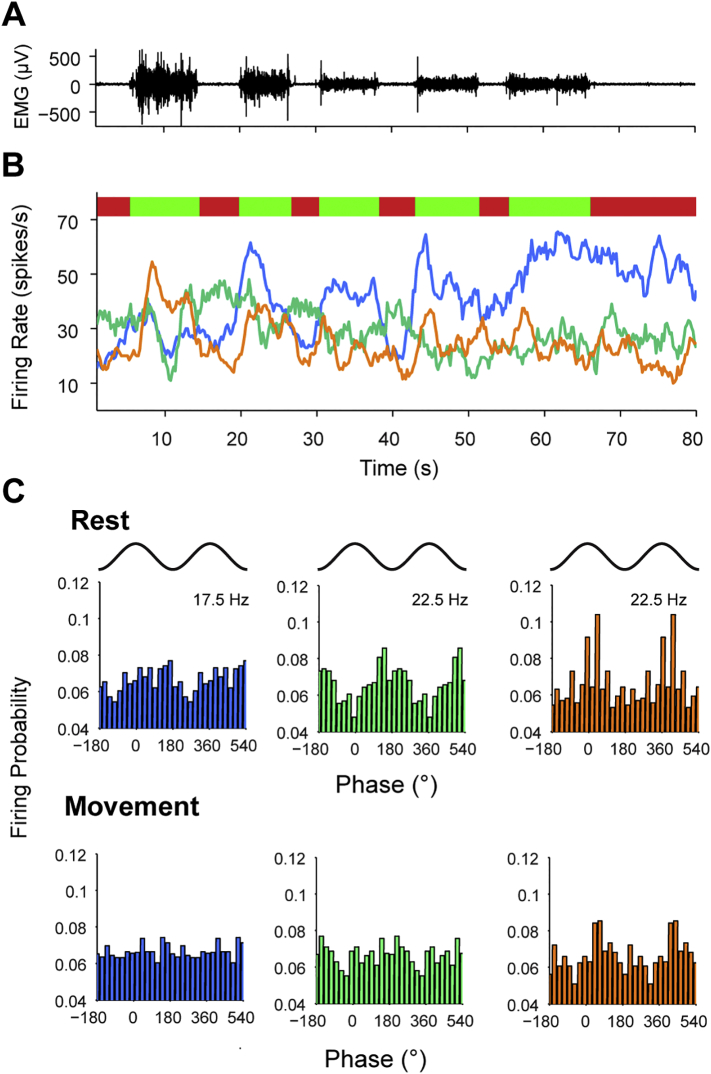
STN units display modulations of both firing rate and cortical phase locking strength during continuous movement. (A) Raw EMG trace of the flexor muscles of the arm contralateral to intracranial recording during intermittent periods of wrist flexion/extension (from Patient 6). (B) Firing rate of three coincidently recorded STN units during the movement periods (green bars) and rest periods (red bars) defined using the flexor EMG. Two of the units (shown by the blue and orange lines) increase their firing rate during the movement periods, while the remaining unit (shown by the green line) fires at higher rates between the movement periods. (C) Phase histograms showing locking to the ECoG calculated for each unit shown in B (colors as in A) across the combined rest and movement periods at the frequency with the strongest phase locking at rest. Two cycles are shown for clarity. For each unit, there is significant phase locking at rest, which decreases during the movement periods. For the first unit (blue), there is no significant phase locking in the movement periods. The other units are desynchronised, but are still phase locked at approximately the same phase as during rest periods.

During movement, STN neurons often display clear modulation of their firing rate (Rodriguez-Oroz et al., 2001, Hanson et al., 2012), whereas STN LFPs become less synchronised with cortical beta oscillations (Lalo et al., 2008, Talakoub et al., 2016). Next we examined whether there was any relationship between these single neuron and network level phenomena. To this end, patients were asked to perform periods of continuous extensions and flexion of the wrist contralateral to the recorded STN, which were monitored using EMG electrodes (Fig. 7A). Rate modulation of STN units could be readily observed online and individual units could both decrease and increase their firing rate during periods of continuous movement (Fig. 7B). A concurrent reduction in the phase locking of individual units could be observed during these during movement periods (Fig. 7C).

**Fig. 8:**
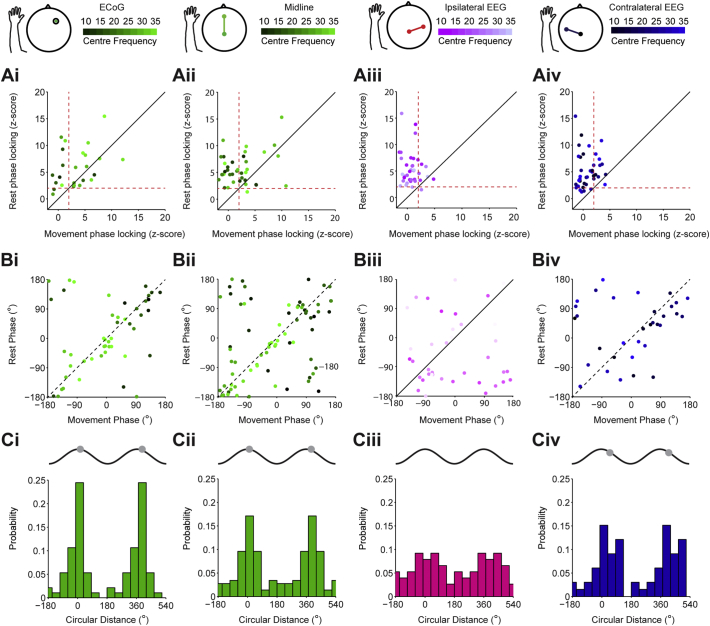
Modulation of cortico-subthalamic phase locking during continuous movement is topographically specific. (Ai–Aiv) The phase locking strength of STN units during rest and movement periods. Each dot shows the phase locking z-score for an STN unit in rest and movement periods at the frequency with the maximum value at rest (frequency displayed using the colour scale above). A z-score value of over 2 is considered significant (indicated by the red dotted lines). For the ECoG (i) and midline EEG (ii) channels, phase-locking strength is generally lower in the rest periods, but several neurons display highly significant locking throughout both conditions. In contrast, for the ipsilateral (iii) and contralateral EEG (iv) channels, most of the units that were significantly phase locked rest are uncoupled (z-score < 2) or have much decreased phase locking values during movement. (B) The mean phase locking angle of all units at all frequencies where significant phase locking phase maintained during rest and movement periods. (Bi–Bii) For the ECoG and midline EEG, the phase locking angles for rest and movement change little and maintain the relationship between phase and frequency (note the move from dark to light coloured dots as phase increases). (Biii–iv) The same plot for units that are significantly phase locked to the ipsilateral EEG shows no real structure. In contrast, the contralateral EEG shows a regression of phase during movement periods. (C) Histograms of circular distance between rest and movement phases shown in B. (Ci–ii) The circular distances are significantly non-uniform for the ECoG and midline EEG (Rayleigh test, *p* < 0.05) and the distances are centred close to 0°, suggesting that there is little change in phase locking angle between rest and movement. (Ciii) The circular distances for the ipsilateral EEG are uniformly distributed (Rayleigh test, *p* > 0.05) and thus the locking angles during rest and movement and unrelated. (Civ) The circular distances for the contralateral EEG are not uniformly distributed (Rayleigh test, *p* < 0.05), but in contrast to the frontal channels, they are centred at 47°, suggesting a phase regression of STN units in relation to these cortical areas during movement.

In order to quantify the relationship between the firing rate response and synchronisation to cortex, we calculated phase locking using a z-score that compared the real vector lengths for each unit to that of ISI shuffled surrogates. This method ensured that synchronisation could not be spuriously related to the number of spikes included in the analysis. For each unit we plotted this phase locking z-score at the frequency with maximum phase locking at rest, against the z-score at the same frequency during movement (Number of units. ECoG *n* = 25, Midline, *n* = 38, Ipsi. *n* = 41, Contra. *n* = 39. Fig. 8Ai–iv). Across all cortical channels, the phase locking of STN units was significantly lower during movement periods (*p* < 0.00000001, F = 107, Df = 1, effect of rest vs. movement, ANOVA). In addition, the phase locking of STN units varied significantly by cortical area (*p* = 0.003, F = 4.85, Df = 3, effect of rest vs. movement, ANOVA). Notably, the phase locking magnitude of STN units to the ECoG during movement was not significantly different to that of any of the cortical channels at rest and was significantly higher than that of the lateral channels (post-hoc Dunn-Sidak test). While the majority of STN units were less synchronised with the ECoG and midline EEG during the movement periods, around half of the units were still significantly phase locked (z-score > 2, ECoG = 60%, Midline = 46%. Fig. 8Aii–Bii). In contrast, relatively few STN units were significantly phase-locked to the ipsilateral and contralateral EEG during movement periods (z-score < 2; ipsilateral, 17%; contralateral, 21%), despite many units having high phase-locking values at rest (Fig. 8Aiii, Aiv). In line with these observations, the proportion of STN units significantly phase locked to frontal channels during movement was significantly higher than for lateral channels (Chi Square, *p* = 0.009).

Several studies have reported beta desynchronization in cortical signals during movement in PD patients (Litvak et al., 2012, Heinrichs-Graham et al., 2014, de Hemptinne et al., 2015, Rowland et al., 2015). If the amplitude of the beta signal was lower during movement periods, the drop in phase-locking strength of a given STN unit could be a consequence of a decrease in the signal-to-noise ratio of the cortical oscillation. However, we found that none of the cortical channels had a significant difference between rest and movement periods in the amplitude of the beta-frequency to which the STN units were phase-locked (Wilcoxon-sign rank test, *p* > 0.05). Moreover, there were no significant correlations between the percentage change in beta amplitude (at which the unit was phase-locked) and the percentage change or difference in z-scored vector length between rest and movement (Pearson Correlation, *p* > 0.05). While these analyses do not preclude cortical desynchronisation per se (which might be detected by triggering to movement onset, combining multiple frequency bands, over shorter timescales etc.), these analyses suggest that it did not underlie the changes in phase locking shown in Fig. 8A.

Next, we examined whether the phase relations between cortex and STN units were preserved across rest and movement. Thus, we could only use STN units that remained significantly locked to a given cortical oscillation during both states at a given frequency (Number of units/Total data points including all frequencies for each neuron: ECoG *n* = 19/54, Midline, *n* = 25/85, Ipsi. *n* = 21/44, Contra. *n* = 22/40. Fig. 8Bi–iv). When the mean rest and movement phase locking angles with the frontal ECoG and midline EEG were plotted against each other, they aligned approximately around the diagonal, suggesting there was little change in the angle of phase locking between states (Fig. Bi, ii). Moreover, the linear phase/frequency relationship was broadly maintained, with locking at lower frequencies closer to the trough and higher frequencies locked to the peak (Fig. Bi, ii). In line with these observations, the circular distances between the phase-locking angles of rest and movement for the ECoG and midline EEG/STN unit pairs were non-uniformly distributed (Fig. 8Ci, Cii, Rayleigh test, *p* < 0.000001), with mean distances close to zero (11.8° and 14°, respectively). In contrast, the phase locking angles of units for ipsilateral EEG channels could be considerably different between rest and movement (Fig. 8Biii) and the circular distance between them was uniformly distributed (Fig. 8Ciii, Rayleigh test, *p* > 0.05). Interestingly, the phase locking angle for the contralateral EEG channel showed a tendency to phase regress around a quarter of a cycle in the movement periods (Fig. 8Biv, Civ). In line with this result, the circular distance between rest and movement was non-uniformly distributed (Rayleigh test, *p* = 0.0003) around a mean distance of 47°, which was significantly different to the mean distances of the ECoG (Watson-Williams test, *p* = 0.00007) and midline EEG (Watson-Williams test, *p* = 0.00005).

In summary, STN neurons widely uncoupled from cortical oscillations during movement and this uncoupling was greater with oscillations in lateral, than in frontal and midline cortical areas. Those STN units remained phase locked to frontal cortical beta oscillations during movement maintained the same phase relations to those signals as at rest.

### 3.5 The magnitude of movement related firing of STN units is correlated with beta desynchronisation

**Fig. 9:**
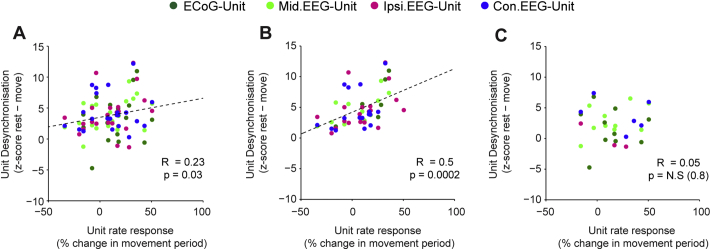
Correlation between movement-related firing rate and beta synchronisation occurs only when units completely desynchronise during movement. The change in firing rate was plotted against the change in cortical synchronisation during movement periods for all units that were significantly synchronised with one of the cortical channels at rest (z-score > 2, cortical channel is denoted by the colour code). Correlations were calculated with Pearson's correlation coefficient, for which R and *p* values are inset. (A) Across all the data, the magnitude of movement-related desynchronisation was weakly, positively correlated with movement-related increase in firing rate. (B) The correlation was stronger using only units that were not significantly phase locked (z-score < 2) to the cortical channels during the movement periods. (C) There was no significant correlation between movement-relate firing rate and movement-related desynchronisation for units that remained phase locked to one of the cortical channels during movement.

Finally, we compared the magnitude of the firing rate response of the single units to their movement-related changes synchronisation to cortical signals (Fig. 9). Overall, there was a positive correlation between the increase in firing rate during movement and the magnitude of desynchronisation with cortex in STN single units (Fig. 9A, Pearson Correlation, *R* = 0.23, *p* = 0.03). We then separated the unit-cortex pairs based on whether they were significantly phase locked during the movement periods (z-score > 2). The relationship between desynchronisation and firing rate was considerably stronger for units that did not remain significantly phase locked during movement (Fig. 9B, Pearson Correlation, *R* = 0.5, *p* = 0.0002). In contrast, for units that remained synchronised to cortical beta during movement periods, there was no correlation between firing rate and desynchronisation (Fig. 9C). The *p*-values for these correlations were significant following FDR correction. These results suggest that firing rate responses are associated with the level of cortical synchronisation, but that this association is lost for units that remain phase locked to cortical oscillations during movement.

### 3.6 The magnitude of cortico-subthalamic synchrony in the high beta range predicts the severity of akinetic/rigid symptoms

**Fig. 10:**
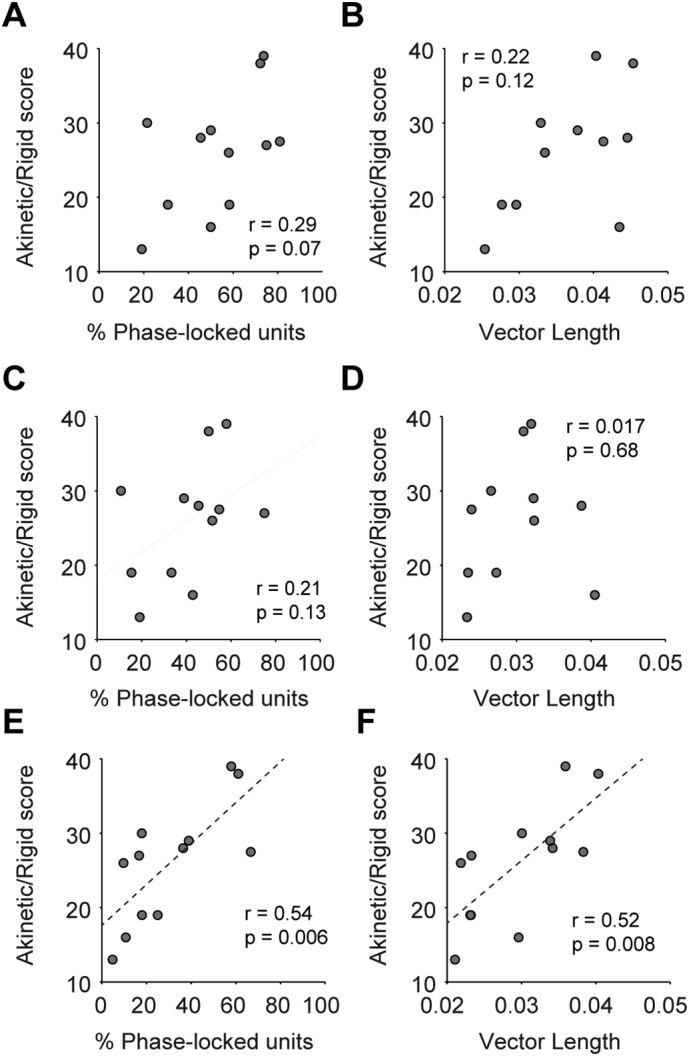
Magnitude of premotor cortico-subthalamic high beta synchronisation is positively correlated with severity of akinetic/rigid symptoms. The percentage of units that were significantly phase locked to the midline EEG and the mean vector length of all units significantly phase locked to the midline EEG were correlated with the pre-operative UPDRS scores for akinesia and rigidity. Phase locking calculations were performed using the all beta frequencies (12–35 Hz, A–B), only the low beta frequencies (12–24 Hz, C, D) and high beta frequencies (25–35 Hz, E, F). Significant positive correlations were observed only at high beta frequencies (E, F) and were significant following FDR correction over all analyses. Dotted lines show linear fits.

Subthalamic beta oscillations are associated with the severity of akinesia/bradykinesia and rigidity, but not tremor and axial symptoms (Kühn et al., 2006, Ray et al., 2008, Sharott et al., 2014). We hypothesised that the strength of synchronisation between premotor cortical activity and STN units, which showed the weakest movement desynchronisation, would also correlate with akinetic/rigid symptoms. As the midline EEG signal was recorded in nearly all patients, we calculated correlations between parameters of STN-unit synchronisation with this channel and the patient's pre-operative UPDRS scores related to akinesia/bradykinesia and rigidity. Neither the percentage of phase locked units nor the mean vector length over the entire beta range were significantly correlated with the pre-operative akinetic/rigid score (Fig. 10A–B). However, when the band was divided in to low (12–22 Hz) and high (25–35 Hz) beta frequencies, both parameters were significantly positively correlated with akinetic/rigid score for high, but not low frequencies (Fig. 10C–F). The *p*-values for these correlations were significant following FDR correction.

## 4 Discussion

In this study, we demonstrate that single STN neurons are most strongly synchronised to beta oscillations in frontal and midline cortical areas with stable time delays that persist during movement. The extent and magnitude of this STN phase locking to beta oscillations recorded over the mesial cortex was highly correlated with the severity of akinetic/rigid motor symptoms. Together, these results show that persistent synchronisation of STN neurons to premotor cortical beta oscillations are a candidate mechanism for bradykinetic symptoms in PD.

### 4.1 Detection of corticosubthalamic synchronisation in Parkinsonian patients

There is no doubt that STN LFPs have provided a crucial window onto subthalamic oscillations in PD. However, because the LFP is a product of synchronous events (Buzsaki et al., 2012), it cannot provide a comprehensive picture of the variance of the firing patterns of individual neurons. Indeed, STN neurons with tremor-, beta- and no oscillatory activity are interspersed side-by-side (Moran et al., 2008, Sharott et al., 2014). Moreover as LFPs likely reflect mainly dendritic events, particularly synaptic inputs (Buzsaki et al., 2012), they cannot provide direct information about the firing rate and/or excitability of underlying neurons in response to cortical oscillations. We sought to exploit single unit recordings to examine these issues in patients and compare these results to the analysis of LFPs.

There are many approaches to analysing synchronisation between neuronal populations. We used a combination of spectral analysis and circular statistics to provide a robust estimate of the temporal relationship between cortical and subthalamic beta oscillations in both LFPs and single units. Our results suggest that analysis of the STN LFP underestimates the influence lateral cortical areas on STN units. The greater sensitivity of the phase locking method employed here, which will capture any bias of the unit to fire on a given phase of the cortical oscillation (even if the spiking skips individual cycles and/or locks to a narrow frequency range), allowed us to define the phase/time delay relationship between STN units and lateral channels. Perhaps most notably, this method allowed us to identify synchronisation of STN units to the contralateral cortex and define the time delay between these signals. Overall, our results suggest that care must be taken in interpreting the communication between cortex and STN using LFPs alone.

### 4.2 Dynamics of corticosubthalamic synchronisation is dependent on cortical area

Recordings of STN LFPs with coincident EEG signals have shown that the strongest corticosubthalamic coherence occurs with mesial areas of cortex (Williams et al., 2002, Fogelson et al., 2006). Source analysis of MEG recordings has demonstrated that this is likely due to the underlying supplementary motor area (SMA), which has highest cortical coupling to STN beta oscillations (Litvak et al., 2010). Here, the midline EEG and ECoG electrodes provided widespread coverage of these cortical areas. Although the placement of the ECoG was over the prefrontal cortex the similarity of the results between this signal and the midline EEG electrode suggests that it may also have picked up this SMA beta source. In this respect, our recordings differed significantly from Shimamoto et al. (2013), where the ECoG strip electrode mostly covered primary sensorimotor cortex. The activity in the lateral EEG channels in our set-up was likely to originate in these cortical areas. Indeed, in both our study and that of Starr and colleagues (Shimamoto et al., 2013), STN neurons tended to fire on the descending phase of the beta oscillation, supporting this premise.

Spectral (Williams et al., 2002, Fogelson et al., 2006) and autoregressive (Cassidy et al., 2002, Lalo et al., 2008, Litvak et al., 2010) analysis methods have been used to demonstrate that, at the level of the LFP, cortical beta oscillations predominantly “lead in phase” over those in the STN. We show that this relationship is also reflected at the level of single units by a frequency dependent linear phase progression of STN units with respect to the cortical oscillation. We calculated a time delay of around 33 ms of STN units in relation to the midline and frontal cortical signals, which is in between the delays previously calculated from the lower and upper beta bands (42 ms and 28 ms, respectively) of midline EEG and STN LFP signals (Fogelson et al., 2006). It has been proposed that this delay reflects transmission through the indirect pathway (Fogelson et al., 2006). However, phase-derived time delays do not necessarily reflect synaptic conduction delays (Aumann and Prut, 2015). Rather, this delay likely reflects the combination of synaptic inputs to STN from different cortical and subcortical inputs that become transiently stable during network beta oscillations (Mallet et al., 2008a, Mallet et al., 2008b, Sharott et al., 2017). Given the limited recording sites in patients, animal models of PD may be better suited to elucidating the exact nature of these interactions. However, the different delays between different cortical areas likely reflects to some extent there relative influence over subthalamic neurons in time. Thus, it is of interest that the ipsilateral EEG, which likely gave the strongest indication of beta oscillations in the primary motor cortex, showed no evidence of leading STN units. This result is consistent with the premotor areas, more involved in movement planning, synchronising with the STN before ipsilateral primary motor areas, primarily involved in movement execution.

Beta oscillations in the contralateral EEG preceded those in the STN units by around double the time delay of the frontal and midline channels. What could be the reason for such a result? STN neurons receive cortical input via the “hyperdirect” pathway and indirect pathway. The monosynaptic hyperdirect pathway is comprised of collateral inputs from axons of pyramidal tract neurons (PTNs) projecting to brainstem and spinal motor centres, as well as other basal ganglia structures (Nambu et al., 2002, Kita and Kita, 2012). The polysynaptic indirect pathway comprises cortical inputs to striatal neurons, which influence STN by inhibiting neurons in the globus pallidus external segment (Albin et al., 1989). The cortical neurons comprising the indirect pathway can be either PTNs or intratelencephalic neurons (ITNs). In contrast to PTNs, ITNs project to both ipsilateral and contralateral striatum (Shepherd, 2013) and therefore have the potential to provide oscillatory input to the contralateral STN through the indirect pathway, the neurons of which can lock to cortical beta oscillations (Sharott et al., 2017). As there is no contralateral PTN input to STN or striatum, contralateral cortex may provide a window on ITN input, which might otherwise be masked by the more active and more oscillatory PTNs (Pasquereau and Turner, 2011). While speculative, this interpretation raises the interesting hypothesis that basal ganglia neurons synchronise to ITNs before PTNs.

### 4.3 Incomplete movement-related beta desynchronisation of STN neurons

The magnitude of STN LFP beta oscillations (Kühn et al., 2004, Doyle et al., 2005, Devos et al., 2006, Tan et al., 2015) and corticosubthalamic oscillatory drive (Lalo et al., 2008) decreases significantly during movement. We demonstrate that individual STN neurons locked to cortical beta oscillations at rest desynchronise from cortical beta oscillations during movement to varying degrees. Importantly, the magnitude of desynchronisation differed across cortical areas. Although locking of STN units to premotor areas was reduced during movement, a large number of units remained synchronised and the maximal firing phase at rest was maintained. This suggests that, during movement, the synaptic interactions underlying the SMA-STN synchronisation are partially maintained, where as those with primary motor areas are mostly absent. This further points to an independence in the coupling of cortico-basal ganglia loops, with areas associated with motor planning retaining stronger oscillatory coupling than those associated with motor execution.

Little is known about how movement related changes in corticosubthalamic synchronisation relate to movement related changes in the firing rate in STN neurons. In healthy primates, neurons in the dorsolateral STN show sharp increases in activity in response to passive limb movements (Wichmann et al., 1994), while in PD patients, STN neurons show rate modulations to a variety of movement parameters (Rodriguez-Oroz et al., 2001, Hanson et al., 2012). We observed gross changes in rate between relatively long continuous movement and rest periods (several seconds). These most commonly consisted of increases in overall firing rate, although movement periods may have contained faster increases and decreases in response to flexion and extension (Rodriguez-Oroz et al., 2001). We found that greater increases in firing rate during these movement periods correlated with the amount of beta desynchronisation from the cortical signals, but only in units that were fully uncoupled from cortical oscillations during the movement periods. It is important to note that many of the units that remained coupled to the frontal/mesial cortex increased their firing rate by up to 50% during movement. Thus, this difference was not due to the units being unresponsive to the movement task, which might indicate that they were in part of the STN that was not concerned with wrist movement. Rather, in a considerable number of STN units, the rate and cortical desynchronisation responses to movement were independent. Given the extensive evidence for beta desynchronisation occurring during healthy movement (Engel and Fries, 2010), this dissociation between firing rate and decoupling from cortical signals could represent a disruption to the normal encoding of movement planning by STN neurons. Specifically, a persistent oscillatory modulation by SMA neurons, which fire strongly during the preparation of movement (Nachev et al., 2008), could prevent the transition of STN neurons from coding the preparation of movement to movement itself.

### 4.4 Clinical relevance of SMA-STN synchronisation in PD?

A pathophysiological role cortical-subthalamic synchronisation was further supported by our finding that its extent was highly correlated with the severity of akinetic rigid symptoms. Previous work has shown that beta desynchronisation following l-dopa (Kühn et al., 2006, Ray et al., 2008, Kühn et al., 2009) and DBS response (Kühn et al., 2008, Zaidel et al., 2010) is correlated with motor improvement. Baseline parameters of beta power and synchrony are also correlated with the preoperative UPDRS score (Chen et al., 2010, Yoshida et al., 2010, Sharott et al., 2014). We extend these observations by showing that such correlations are also present at the level of corticosubthalamic syncrhonisation. Taken together with the finding that beta oscillations in STN neurons are driven by premotor cortical oscillations, correlations between motor impairment and local STN synchronisation may even be a proxy of this cortico-basal ganglia coupling.

Whether or not it represents a primary pathological process, cortico-subthalamic beta synchronisation provides important clues as to the way in which different cortical areas influence the firing of STN neurons in PD patients. In particular, our results demonstrate that several cortical areas modulate the firing pattern of STN neurons through rest and movement states. The sustained nature of this synchronisation could represent reverberation in the cortico-basal ganglia-thalmo cortical loop that would usually be prevented by the resistance of many basal ganglia neuron populations to being entrained by cortical oscillations (Raz et al., 2000, Magill et al., 2004). Recently, there has been much interest in the idea of closed-loop deep brain stimulation for the treatment of akinetic-rigid symptoms of PD (Beuter et al., 2014, Little and Brown, 2014, Arlotti et al., 2016, Kern et al., 2016, Rossi et al., 2016). The utility of such an approach has been demonstrated in MPTP-treated primates (Rosin et al., 2011), where stimulation of basal ganglia triggered with a fixed time delay by oscillating cortical spikes was substantially more effective than continuous stimulation. As time delays between premotor cortex and STN are stable between rest and movement, our results provide further evidence of the potential of such approaches for designing novel stimulation paradigms to achieve more efficacious treatment of akinetic/rigid symptoms in PD.

The following are the supplementary data related to this article.

Supplemental Table 1 Patient details. Supplemental Table 1
