# Supplementary material for: Spatio-temporal dynamics of cortical drive to human subthalamic nucleus neurons in Parkinson's disease
Source: Neurobiol Dis. 2018 Apr;112:49–62. doi: 10.1016/j.nbd.2018.01.001 (PMC5821899; doi:10.1016/j.nbd.2018.01.001)
Supplement: Supplemental Table 1 — Patient details. [file mmc1.pdf]

Case	Age (Yrs) and Sex	Disease Duration (Yrs)	Motor UPDRS OFF	Motor UPDRS ON	Medication pre-operation	Hoehn/ Yahr Score	Dominant side	Major Symptoms	Hemispheres analysed	ECoG
1	61F	25	50	30	Levodopa 700 mg<br>Carbidopa 150 mg<br>Entacapone 1000 mg<br>Benserazide 25 mg<br>Pramipexol 0.26 mg	5	Left	Tremor,<br>Akinesia/rigidity<br>Fluctuations	Right	Yes
2	62M	14	33	16	Amantadine 300 mg<br>Levodopa 900 mg<br>Benserazide 50 mg<br>Entacapone 1400 mg<br>Carbidopa 150 mg<br>Rasagiline 1 mg	3	Tremor right/<br>Akinesia and rigidity left	Fluctuations<br>Dyskinesia<br>Tremor<br>Akinesia/rigidity	Left	Yes
3	72F	18	41	19	Levodopa 700mg<br>Carbidopa 175mg	4	Left	Equivalence type Fluctuations,<br>Dyskensia	Left	Yes
4	70F	8	38	21	Ropinirole 8mg<br>Alpha-dihydroergocryptine 40 mg<br>Amantadine 600 mg<br>Levodopa 250mg<br>Carbidopa 150mg	3	Left	Bradykinesia<br>Fluctuations<br>Dyskinesia	Both	No
5	69F	16	54	20	Levodopa 600 mg<br>Carbidopa 100 mg<br>Pramipexol 2.8 mg<br>Entacapone 800 mg<br>Amantadine 200 mg	4	Right	Tremor<br>Dyskinesias.	Left	No
6	67M	25	55	19	Levodopa 1250 mg<br>Entacapone 1400 mg	3	Right	Equivalence<br>Fluctuations	Both	Yes

					Carbidopa 312.5mg<br>Rotigotine 6 mg<br>Amatadine 150mg			Dyskinesia		
7	64M	15	53	39	Amantadine 300 mg<br>Levodopa 550 mg<br>Ropinirole 20 mg<br>Entacapone 900 mg<br>Carbidopa 112.5 mg<br>Benserazide 25 mg	4	Left	Akinesia/rigidity<br>Camptocormia	Left	Yes
8	69F	9	21.5	7	Levodopa 450 mg<br>Lisuride 0.9 mg<br>Rotigotine 4 mg<br>Amantadine 300 mg	3	Right	Equivalence type<br>Fluctuations<br>Dyskinesia	Both	Yes
9	66M	11	28	14	Amantadine 150 mg<br>Levodopa 1450 mg<br>Tolcapone 300mg<br>Carbidopa 312.5<br>Benserazide 50mg	4	Right	Akinesia/rigidity<br>Fluctuations	Both	No
10	63F	17	35	16	Rotigotine 4mg<br>Ropinirole 25mg<br>Levodopa 350mg	3	Left	Akinesia/rigidity<br>Bradykinesia<br>Fluctuations<br>Dyskinesia	Both	Yes
11	51F	21	21	12	Levodopa 125 mg<br>Cabergoline 4 mg<br>Pramipexole 7 mg	3	Right	Fluctuations<br>Dyskinesias	Both	No

12	67,M	10	19	4	Entacapone 1200 mg<br>Levodopa 700 mg<br>Carbidopa 175 mg	3	Right	Bradykinesia<br>Rigidity<br>Fluctuations	Right	No

Supplemental Table 1. Patient Details
